# Nutrition knowledge, label use, and dietary diversity among a sample of university students in Bangladesh: a cross-sectional investigation

**DOI:** 10.1017/jns.2026.10098

**Published:** 2026-05-14

**Authors:** M. M. Mehedi Hasan, Md. Hasan Al Banna, Trisha Mallick, Keith Brazendale, Najim Z. Alshahrani, Humayra Alam Mouly, Farzana Afroz, Sumaia Sahrin

**Affiliations:** 1 Department of Human Nutrition and Dietetics, Faculty of Nutrition and Food Science, Patuakhali Science and Technology University, Patuakhali, Bangladesh; 2 Faculty of Nutrition and Food Science, Patuakhali Science and Technology University, Patuakhali, Bangladesh; 3 Department of Environmental Sanitation, Faculty of Nutrition and Food Science, Patuakhali Science and Technology University, Patuakhali, Bangladesh; 4 Department of Health Sciences, University of Central Florida, Orlando, USA; 5 Department of Family and Community Medicine, Faculty of Medicine, University of Jeddah, Jeddah, Saudi Arabia; 6 Department of Public Health, North South University, Dhaka, Bangladesh; 7 Department of Biochemistry and Food Analysis, Faculty of Nutrition and Food Science, Patuakhali Science and Technology University, Patuakhali, Bangladesh

**Keywords:** Bangladesh, Dietary diversity, Nutrition knowledge, Nutrition label use, Students

## Abstract

The objective of this study was to assess the prevalence and predictors of nutrition knowledge, nutrition label use and dietary diversity among a sample of university students in Bangladesh. A cross-sectional study was carried out from November 2023 to April 2024 among undergraduate and postgraduate students. About 428 participants (aged 18–26 years) completed a structured questionnaire containing questions related to their demographic information, nutrition knowledge, nutrition label use, and dietary diversity (consumption of different food categories). Separate logistic regression models identified the main factors associated with nutrition knowledge, nutrition label use and dietary diversity. Approximately 32.5% participants had satisfactory nutrition knowledge and 36.0% were frequent nutrition label users. Female participants were more likely to have satisfactory nutrition knowledge (adjusted odds ratio, AOR = 2.05, 95% CI = 1.29, 3.26) and use nutrition labels more frequently (AOR = 1.97, 95% CI = 1.22, 3.17) than their male counterparts respectively. Around 40% of the participants had a high level of dietary diversity. Students with satisfactory nutrition knowledge (AOR = 3.02, 95% CI = 1.41–6.45) and frequent use of nutrition labels (AOR = 2.73, 95% CI = 1.35–5.55) had a higher dietary diversity compared to their counterparts. Less than half of Bangladeshi students in this study have adequate nutrition knowledge, label use, and dietary diversity. The findings highlight the importance of implementing nutrition awareness programmes and nutrition education interventions for university students, such as basic food and nutrition knowledge and the use of nutrition labels, to enhance their dietary diversity and health status.

## Introduction

Many factors influence life quality, but nutrition stands out as one of the most significant. A balanced diet that includes foods abundant in fat, protein, carbohydrates, vitamins, and minerals may be achieved with the aid of nutrition education. Nutritional knowledge and dietary habits are essential for the maintenance of a healthy lifestyle, particularly for young individuals. Unhealthy weight gain and inadequate nutrition are often associated with the rapid changes and transitions that young adults experience in their lives.^(1,2)^ Individuals’ nutrition knowledge, nutrition label use and dietary diversity are essential for maintaining healthy dietary habits and promoting overall health. Previous studies on the relationship between dietary habits and nutrition knowledge have found that people with limited knowledge often overlook reading nutrition labels, struggle to understand the information on the label, and misinterpret the amount of food needed for a balanced diet.^(3–5)^

Nutrition knowledge may be referred to as the individual cognitive process in relation to information on food and nutrition that can influence food choices.^(6)^ Moreover, individual nutrition knowledge can encourage the consumption of nutritious foods and, in turn, encourage dietary changes that can reduce the risk of acquiring non-communicable diseases such as obesity.^(7,8)^ The term ‘dietary diversity’ refers to the variety of foods consumed throughout a particular period of time. Consuming a diverse diet is the most effective way to ensure nutrient adequacy, as it enables individuals to source a range of macro and micronutrients.^(9)^ Previous studies have shown that low nutrition knowledge has been linked to dietary habits that eventually result in obesity.^(10)^

Nutrition labelling serves as a key tool to facilitate healthier choices by making nutritional information accessible, thereby supporting individuals who are motivated to adhere to healthy eating habits.^(11)^ To be effective, nutritional labels must provide precise, consistent, and comprehensible details about the contents of food.^(12)^ Many countries have implemented laws mandating the inclusion of nutrition labelling on all prepackaged foods and restaurant menus, as it is seen as an essential educational and beneficial tool for promoting more nutritious food choices^(13)^ and has been linked to improved diet quality.^(14)^ Studies show that individuals who read the nutrition label on prepackaged foods have a diet with more fibre, fruits, vegetables, and legumes, and lower sodium, than people who don’t read the nutrition label.^(3,15)^

Previous studies of Bangladeshi adults have reported moderate levels of nutrition literacy, and have reported associations between healthy eating behaviours and nutrition literacy.^(16,17)^ According to research, customers who demonstrate adequate nutrition knowledge are more prone to use nutritional labels.^(18)^ Nutrition initiatives and strategies in South Asia, including in Bangladesh, exhibit significant disparities and often lack coherent preventive strategies.^(19)^ Specifically, the focus within Bangladesh’s healthcare system remains skewed toward treating existing conditions rather than implementing preventive measures such as early nutritional intervention and public health education programmes.^(20,21)^ Prior research has shown that adequate knowledge about food and nutrition is associated with healthier food choices, cooking skills, eating habits, and diet quality.^(22)^ However, it has been challenging for nutrition experts, health practitioners, and stakeholders in Bangladesh to generate and implement health promotion interventions which would enhance dietary patterns of the Bangladeshi population.

### Rationale and objective of this study

In Bangladesh, food-related disparities exist in eating behaviour and nutrient intake patterns. A significant number of individuals aged 18 years and older have a chronic illness and are overweight or obese as a consequence of malnutrition.^(23,24)^ University students, or emerging adults, are more likely to develop unhealthy eating habits and become overweight or obese as they often away from family during this unique time of their lives.^(25)^ At this stage, getting sufficient nourishment is essential for maintaining physical and mental health and promoting healthy brain and intellectual growth.^(26,27)^ Encouraging youth and university learners to acquire sufficient knowledge about nutrition and develop habits of reading nutrition labels might be an essential initiative towards maintaining good health, as it could significantly influence their future food preferences, improve diet quality, and potentially reduce the medical burden placed on the country.^(25,28)^

The existing Bangladesh-based studies have examined these components (nutrition knowledge, nutrition label use, and dietary diversity) separately without an integrated approach. For instance, prior research has documented the prevalence of low dietary diversity,^(29,30)^ assessed general nutrition knowledge and food habits,^(31,32)^ and explored food label knowledge and practices.^(33)^ Other studies have investigated distinct psychological or behavioural constructs, such as food addiction, orthorexia nervosa, stress, and nutrition literacy in relation to eating behaviours.^(34,35)^ Nonetheless, two critical gaps remain. First, an integrated evaluation of these three core determinants within a single analytical framework is absent. This fragmentation means that despite the recognized importance of nutrition knowledge and label use, their combined role in shaping dietary outcomes, such as dietary diversity, remains unexplored in Bangladesh. This leads directly to the second gap: evidence for the predictive role of nutrition knowledge and nutrition label use on dietary diversity among Bangladeshi young adults is entirely lacking. Therefore, this study aims to fill these gaps by conducting an integrative analysis of nutrition knowledge, nutrition label use, and dietary diversity in a large sample of university students in Bangladesh.

Specific objectives are to: i) assess the prevalence and correlates of nutrition knowledge and nutrition label use among the participants, and ii) evaluate dietary diversity and its predictors, specifically the predictive role of nutrition knowledge and nutrition label use.

## Materials and methods

### Study design and ethics

This cross-sectional study was carried out at four public universities in Bangladesh from November 2023 to April 2024 among undergraduate and postgraduate students. These universities were purposefully selected, to facilitate data collection, from three distinct divisions of Bangladesh. Two universities were chosen from the Barishal division and the other two from the Dhaka and Chattogram divisions. Conducting research across a larger number of institutions would require considerably more time, resources, logistical support and coordination, which was beyond the scope of this study.

The Institutional Ethical Committee of Patuakhali Science and Technology University, Bangladesh (reference number: PSTU/IEC/2023/74) reviewed and approved the research protocol. All participants signed informed consent forms after receiving details about the study. Participation was voluntary and all data were de-identified and kept completely confidential.

### Study eligibility criteria

To be eligible for the study, potential participants had to be an adult (age ≥18), a native of Bangladesh, and a current university student. Students with severe mental or physical health conditions were excluded from this study.

### Sample size estimation

The sample size was determined using the Cochran formula. The following factors were taken into consideration for estimating the sample size: (i) a 50% predictive prevalence (*p* = 0.5), (ii) a 95% confidence level (*Z* = 1.96), and (iii) a 5% margin of error (*e* = 0.05).

The minimum sample size for this study was, 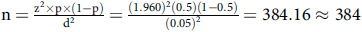


The minimal sample size needed was 384, and the study team sampled higher than this number to account for missing or incomplete data.

### Sampling and survey procedures

The study team approached groups of students by visiting popular locations where students typically gather, such as social areas and canteens, and informed them of study information and extended invitations to participate. Participants were selected using a simple random sampling method, and data collection was conducted through face-to-face interviews by trained data collectors. Interviews were conducted individually, typically immediately following recruitment. Participant recruitment continued until the pre-determined minimum sample size was reached. Although participants’ response numbers were not systematically recorded, the target sample size was exceeded due to a positive level of interest. Field notes indicated that non-participation was mainly due to time constraints. Four data collectors conducted 12-to-15-minute interviews with participants from the four universities (purposively selected). An online training session via Zoom was organized by the principal investigator to instruct data collectors on participant eligibility, effective interview techniques, and interview scheduling.

### Variables and measures

Participants’ demographics and descriptive characteristics were collected via questionnaire including sex, age, academic level, study subject, living status, family income, self-reported physical activity level and self-reported BMI (all independent variables). BMI was not calculated from self-reported height and weight; instead, participants directly reported their perceived weight status as underweight, normal weight, or overweight/obese. Moreover, participants’ nutrition knowledge, nutrition label use and dietary diversity were assessed as primary outcome variables (described below).

#### Assessment of nutrition knowledge

The validated consumer nutrition knowledge scale (CoNKS) developed by Dickson-Spillmann et al. (2011)^(36)^ was used to assess nutrition knowledge. This tool includes questions on both declarative knowledge (such as calorie content, nutrient composition, and food comparisons) and procedural knowledge (such as the health benefits of food groups, fats, fruits, and vegetables). The scale consists of 20 questions with ‘yes’, ‘no’, and ‘don’t know’ response options to evaluate these aspects of nutritional knowledge. The CoNKS has been used in several quantitative studies to examine nutrition knowledge.^(37,38)^

Scores can range from 0 to 20, with each correct answer earning one point and incorrect answers receiving zero. Using a modified Bloom’s taxonomy, scores were categorized as follows: 16–20 points (80–100%) were classified as good, 10–15 points (50–79%) as moderate, and below 10 points (<50%) as poor. Additionally, scores were grouped into satisfactory (≥16 points) and unsatisfactory (<16 points) categories.^(37)^

#### Nutrition label use

The use of nutrition information on food labels before purchasing foods/beverages was assessed by asking a single question, in accordance with previous research.^(39)^ The question ‘How often do you read the nutrition labels on food labels before purchasing foods or beverages?’ has four answer options: ‘never or rarely’, ‘sometimes’, ‘often’, and ‘always or almost always’. Students who chose often, always, or almost always were classified as frequent nutrition label users.^(39)^

#### Assessment of dietary diversity

The dietary diversity was measured using a questionnaire that follows the Food and Agriculture Organization guidelines and focuses on the consumption of nine major food groups in past 24 h.^(9)^ These food groups are: (i) cereals, (ii) dark green, leafy vegetables, (iii) vitamin A-rich fruits and vegetables, (iv) other fruits and vegetables, (v) organ meat, (vi) meat, fish, and seafood, (vii) eggs, (viii) legumes, nuts, and seeds, and (ix) milk and milk products. Low dietary diversity was defined as consuming ≤3 food groups, moderate dietary diversity as consuming 4 to 5 food groups, and high dietary diversity as consuming ≥6 food groups.^(40)^

### Data analysis

Survey data were analysed using STATA (version 16). Numbers of responses, percentages, mean and standard deviations were calculated. A chi square test examined the association between the outcome (nutrition knowledge, nutrition label use and dietary diversity) and predictor variables.

Separate adjusted binary logistic regression models were fitted to identify the associated factors of satisfactory nutrition knowledge and frequent nutrition label use. Furthermore, multinomial logistic regression analysis was applied to identify the predictors of medium and high dietary diversity level considering low dietary diversity as a reference group. Before running the adjusted regression model, multicollinearity of independent variables were checked using the variance inflation factor, and the findings found no evidence of collinearity. Each adjusted regression model met the Hosmer and Lemeshow model fitness criteria. The results of the logistic regression analyses were reported as odds ratios with corresponding 95% CIs. *P* values of less than 0.05 were considered statistically significant.

## Results

### Study participants characteristics

In total, 428 participants provided complete data. Approximately half of participants were male (50.7%) and ranged in age from 18 to 26 years [mean age: 23 years, SD: 1.25]. Most participants reported studying at the undergraduate level (76.2%) and residing on the university campus (74.5%). Half of the participants’ families were middle income class (49.5%). Nearly half of the participants (48.1%) reported low levels of physical activity (almost inactive) and 75% reported a normal body weight (Table 1).

**Table 1.** Study participants’ demographic and descriptive information (*n* = 428)

Variables	Frequency	Percent
Sex		
Male	217	50.7
Female	211	49.3
Age (years)		
18 to 20	138	32.2
21 to 22	130	30.4
23 to 26	160	37.4
Academic level		
Undergraduate	326	76.2
Post-graduate	102	23.8
Study major		
Engineering	56	13.1
Health science	124	29.0
Biological science	106	24.8
Business studies	58	13.6
Others	84	19.6
Living status		
Living university hall	319	74.5
Rented house with friends	82	19.2
Living with family	27	6.3
Family income class		
Lower	32	7.5
Middle	212	49.5
Upper	184	43.0
Self-reported physical activity level		
Almost inactive	206	48.1
Moderate	146	34.1
Regular	76	17.8
Self-reported BMI		
Underweight	45	10.5
Normal weight	323	75.5
Overweight/obese	60	14.0

### Prevalence and factors of nutrition knowledge and nutrition label use

Table 2 presents the results related to nutrition knowledge and nutrition label use. Approximately two-thirds of the participants (*n* = 289, 67.5%) exhibited unsatisfactory nutrition knowledge, and 36.0% of participants (*n* = 154) were frequent users of nutrition labels.

**Table 2.** Predictors of satisfactory nutrition knowledge among Bangladeshi university students

	Nutrition knowledge			Adjusted regression model		
Variables	Unsatisfactory	Satisfactory	*P*	AOR	95%CI	*P*
Sex	–	–	**0.004**	–	–	–
Male	161 (74.2)	56 (25.8)	–	RC.	–	–
Female	128 (60.7)	83 (39.3)	–	2.05	1.29, 3.26	**0.003**
Age (years)	–	–	0.950	–	–	–
18 to 20	93 (67.4)	45 (32.6)	–	RC.	–	–
21 to 22	89 (68.5)	41 (31.5)	–	0.99	0.58, 1.71	0.990
23 to 26	107 (66.9)	53 (33.1)	–	1.16	0.69, 1.93	0.564
Academic level	–	–	0.785	–	–	–
Undergraduate	219 (67.2)	107 (32.8)	–	RC.	–	–
Post-graduate	70 (68.6)	32 (31.4)	–	0.93	0.56, 1.54	0.764
Study major	–	–	0.346	–	–	–
Engineering	34 (60.7)	22 (39.3)	–	1.62	0.78, 3.32	0.189
Health science	79 (63.7)	45 (36.3)	–	1.31	0.74, 2.34	0.355
Biological science	76 (71.7)	30 (28.3)	–	RC.	–	–
Business studies	38 (65.5)	20 (34.5)	–	1.46	0.71, 3.00	0.302
Others	62 (73.8)	22 (26.2)	–	0.71	0.36, 1.39	0.322
Living status	–	–	0.657	–	–	–
Living university hall	212 (66.5)	107 (33.5)	–	1.11	0.44, 2.79	0.832
Rented house with friends	57 (69.5)	25 (30.5)	–	0.99	0.36, 2.75	0.983
Living with family	20 (74.1)	7 (25.9)	–	RC.	–	–
Family income class	–	–	**0.007**	–	–	–
Lower	21 (65.6)	11 (34.4)	–	RC.	–	–
Middle	158 (74.5)	54 (25.5)	–	0.68	0.29, 1.54	0.351
Upper	110 (59.8)	74 (40.2)	–	1.27	0.56, 2.86	0.567
Physical activity level	–	–	0.753	–	–	–
Almost inactive	137 (66.5)	69 (33.5)	–	RC.	–	–
Moderate	102 (69.9)	44 (30.1)	–	1.02	0.63, 1.67	0.929
Regular	50 (65.8)	26 (34.2)	–	1.27	0.69, 2.32	0.431
BMI	–	–	0.685	–	–	–
Underweight	32 (71.1)	13 (28.9)	–	0.81	0.39, 1.65	0.568
Normal weight	219 (67.8)	104 (32.2)	–	RC.	–	–
Overweight/obese	38 (63.3)	22 (36.7)	–	1.22	0.66, 2.25	0.507

*Note*: RC. refers to reference category of independent variables; AOR, adjusted odds ratio; *P*, probability value. Bolded values indicate statistically significant at *p* < 0.05. Hosmer–Lemeshow: Chi-square = 9.42, *p* = 0.3085.

Bivariate analysis found participants’ gender (*p* = 0.004) and family income class (*p* = 0.007) were significantly correlated with the level of nutrition knowledge (satisfactory or unsatisfactory). According to our adjusted regression analysis, female participants were more likely to have satisfactory nutrition knowledge than their male counterparts (adjusted odds ratio, AOR = 2.05, 95% CI = 1.29, 3.26, *p* = 0.003) (Table 2).

The chi-square test shows that participants’ sex (*p* = 0.001) and nutrition knowledge level (*p* < 0.001) were significantly associated with nutrition label use (frequent user vs. infrequent user). The adjusted regression model demonstrated that female participants reported using nutrition labels more frequently compared to male counterparts (AOR = 1.97, 95%CI = 1.22, 3.17, *p* = 0.005). Moreover, participants who had satisfactory nutrition knowledge were more likely to use nutrition labels than those who had unsatisfactory nutrition knowledge (AOR = 3.55, 95% CI = 2.26, 5.55, *p* < 0.001) (Table 3).

**Table 3.** Predictors of frequent nutrition label use among university students in Bangladesh

	Nutrition label use			Adjusted model		
Variables	Infrequently	Frequently	*P*	Odds ratio	95%CI	*P*
Sex	–	–	**0.001**	–	–	–
Male	156 (71.9)	61 (28.1)	–	RC.	–	–
Female	118 (55.9)	93 (44.1)	–	1.97	1.22, 3.17	**0.005**
Age (years)	–	–	0.383	–	–	–
18 to 20	88 (63.8)	50 (36.2)	–	RC.	–	–
21 to 22	89 (68.5)	41 (31.5)	–	0.84	0.49, 1.47	0.549
23 to 26	97 (60.6)	63 (39.4)	–	1.32	0.79, 2.19	0.295
Academic level	–	–	0.435	–	–	–
Undergraduate	212 (65.0)	114 (35.0)	–	RC.	–	–
Post-graduate	62 (60.8)	40 (39.2)	–	1.10	0.66, 1.83	0.711
Study major	–	–	0.160	–	–	–
Engineering	41 (73.2)	15 (26.8)	–	0.56	0.26, 1.21	0.143
Health science	70 (56.5)	54 (43.5)	–	1.09	0.61, 1.93	0.780
Biological science	67 (63.2)	39 (36.8)	–	RC.	–	–
Business studies	37 (63.8)	21 (36.2)	–	1.01	0.49, 2.07	0.988
Others	59 (70.2)	25 (29.8)	–	0.61	0.31, 1.19	0.149
Living status	–	–	0.355	–	–	–
Living university hall	198 (62.2)	121 (37.9)	–	1.11	0.43, 2.86	0.823
Rented house with friends	57 (69.5)	25 (30.5)	–	0.89	0.32, 2.53	0.833
Living with family	19 (70.4)	8 (29.6)	–	RC.	–	–
Family income class	–	–	0.373	–	–	–
Lower	22 (68.8)	10 (31.2)	–	RC.	–	–
Middle	141 (66.5)	71 (33.5)	–	1.29	0.54, 3.12	0.559
Upper	111 (60.3)	73 (39.7)	–	1.41	0.58, 3.42	0.435
Physical activity level	–	–	0.879	–	–	–
Almost inactive	134 (65.0)	72 (35.0)	–	RC.	–	–
Moderate	93 (63.7)	53 (36.3)	–	1.27	0.77, 2.09	0.353
Regular	47 (61.8)	29 (38.2)	–	1.39	0.75, 2.58	0.294
BMI	–	–	0.835	–	–	–
Underweight	27 (60.0)	18 (40.0)	–	1.19	0.60, 2.38	0.609
Normal weight	208 (64.4)	115 (35.6)	–	RC.	–	–
Overweight/obese	39 (65.0)	21 (35.0)	–	0.86	0.45, 2.38	0.631
Nutrition knowledge	–	–	**<0.001**	–	–	–
Satisfactory	60 (43.2)	79 (56.8)	–	3.55	2.26, 5.55	**<0.001**
Unsatisfactory	214 (74.0)	75 (26.0)	–	RC.	–	–

*Note*: RC. refers to reference category of independent variables. *P,* probability value. Bolded values indicate statistically significant at *p* < 0.05. Hosmer–Lemeshow test: Chi-square = 10.57, *P* = 0.2274.

### Dietary diversity and its predictors

**Figure 1.**
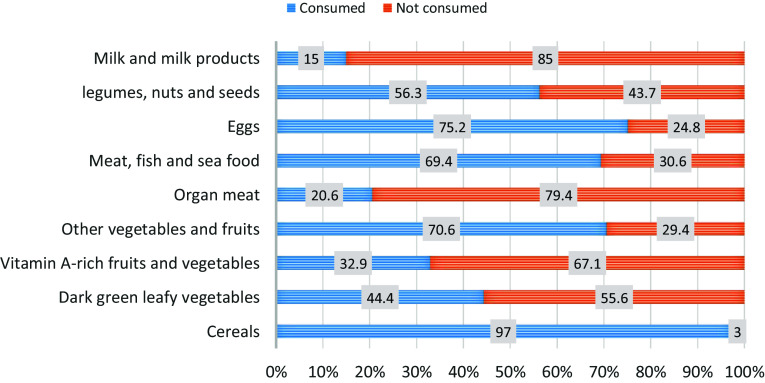
The consumption of different food groups by Bangladeshi university students in the last 24 hours.

Approximately 40% of the participants had a high level of dietary diversity (*n* = 168, 39.3%), and 42.2% (*n* = 181) and 18.5% (n = 79) had medium and low levels of dietary diversity, respectively. Figure 1 represents the percentage of surveyed university students who consumed different food groups in the previous 24 hours. Cereals (97.0%), eggs (75.2%) and other vegetables and fruits (70.6%) were the top three consumed food groups. The lowest consumed food group was milk and milk products (15%), followed by organ meat (20.6%) and vitamin A-rich fruits and vegetables (32.9%) (Figure 1).

Adjusted multinomial regression analysis shows that participants with satisfactory nutrition knowledge had a higher dietary diversity compared to their counterparts (AOR = 3.02, 95% CI = 1.41-6.45, *p* = 0.004). Moreover, participants who were frequent nutrition label users had a higher level of dietary diversity compared to those who were not frequent nutrition label users (AOR = 2.73, 95%CI = 1.35-5.55, *p* = 0.005) (Table 4).

**Table 4.** Adjusted multinomial regression analysis demonstrating the factors associated with dietary diversity among university students in Bangladesh

	Medium level of dietary diversity		High level of dietary diversity	
Variables	Odds ratio [95% CI]	*p*	Odds ratio [95% CI]	*P*
Sex				
Male	Reference	–	Reference	–
Female	1.63 [0.85–3.09]	0.140	1.19 [0.61–2.27]	0.606
Age (years)				
18 to 20	Reference	–	Reference	–
21 to 22	0.42 [0.20–0.86]	0.018*	0.51 [0.25–1.06]	0.071
23 to 26	1.05 [0.51–2.15]	0.904	0.86 [0.41–1.79]	0.684
Academic level				
Undergraduate	Reference	–	Reference	–
Post-graduate	1.69 [0.81–3.48]	0.157	1.47 [0.70–3.07]	0.307
Study major				
Engineering	1.09 [0.39–2.96]	0.872	1.08 [0.39–2.97]	0.876
Health science	0.81[0.37–1.77]	0.592	0.70 [0.32–1.55]	0.381
Biological science	Reference	–	Reference	–
Business studies	2.45 [0.82–7.31]	0.107	1.39 [0.44–4.34]	0.574
Others	0.47 [0.20–1.10]	0.082	0.74 [0.33–1.70]	0.483
Living status				
Living university hall	0.66 [0.20–2.17]	0.499	1.40 [0.39–5.10]	0.608
Rented house with friends	0.31 [0.08–1.09]	0.069	0.72 [0.18–2.83]	0.635
Living with family	Reference	–	Reference	–
Family income class				
Lower	Reference	–	Reference	–
Middle	0.70 [0.22–2.26]	0.547	0.57 [0.18–1.81]	0.337
Upper	1.18 [0.35–3.95]	0.783	0.89 [0.27–2.95]	0.853
Physical activity level				
Almost inactive	Reference	–	Reference	–
Moderate	0.77 [0.40–1.49]	0.440	0.74 [0.38–1.43]	0.369
Regular	1.59 [0.65–3.87]	0.306	1.82 [0.76–4.38]	0.181
BMI				
Underweight	1.58 [0.57–4.39]	0.378	1.50 [0.53–4.27]	0.447
Normal weight	Reference	–	Reference	0.408
Overweight/obese	1.21 [0.49–2.94]	0.670	1.44 [0.60–3.47]	–
Nutrition knowledge				
Satisfactory	2.28 [1.06–4.91]	0.035*	3.02 [1.41–6.45]	0.004*
Unsatisfactory	Reference	–	Reference	–
Nutrition label use				
Infrequent user	Reference	–	Reference	–
Frequent user	1.93 [0.95–3.93]	0.071	2.73 [1.35–5.55]	0.005*

*Note*: Bolded and asterisk values represent statistically significant at *p* < 0.05. Base category = low dietary diversity.

## Discussion

The current study aimed to investigate the status and predictors of nutrition knowledge, nutrition label use and dietary diversity among a sample of university students in Bangladesh. The results of this study indicate that participants reporting satisfactory nutrition knowledge and frequent nutrition label users had a higher level of dietary diversity. Additionally, female participants were more likely to have satisfactory nutrition knowledge and use nutrition labels more frequently than their male counterparts. The current study findings highlight the importance of nutrition knowledge and nutrition labels and their association with dietary diversity among university students in Bangladesh.

Our study found that most of the participants showed unsatisfactory levels of nutritional knowledge (67.5%) which is similar to previous studies conducted in Bangladesh,^(28)^ India,^(41)^ Ethiopia,^(42)^ and Arab countries.^(37)^ Research from several countries demonstrated that school-based nutrition and health education may significantly increase students’ knowledge of nutrition and enable them to adopt healthy eating behaviours.^(43)^ While this subject is typically studied exclusively in nutrition-related departments at the university level, the current study expanded on this by including respondents from a wide range of academic backgrounds to better explore nutrition knowledge across the general student population.

This study also revealed that most of the students were infrequent nutrition labels users which is consistent with previous studies conducted in Malaysia.^(44)^ Individuals who frequently use nutrition labels tend to possess more satisfactory nutrition knowledge compared to infrequent users. Consistent with our study, previous studies conducted among university students in the US discovered a positive association between nutrition knowledge and the use of nutrition labels,^(18,45)^ and other studies have reported individuals with lower levels of nutrition knowledge are less likely to use nutrition labels.^(10,46)^ This may be explained by the fact that individuals who possessed a greater degree of nutritional literacy utilized nutritional labels more frequently, primarily due to their capacity to assess and comprehend the information on food labels. Additionally, consumers who were more proficient in obtaining information from nutritional labels were more likely to apply them.^(47)^ Our results provide important implications about the potential utility of nutrition programmes that specifically focus on university students and young adults. Programmes or workshops that raise the awareness and education around nutrition knowledge, including how to read, understand, and incorporate nutrition labels in to the day-to-day lives of young adult populations/students may be one effective strategy to improve dietary diversity and overall healthy eating habits.

Another notable finding revealed female participants had higher satisfactory nutritional knowledge (39.3%) and use of nutritional labels (44.1%) compared to male counterparts. These findings are consistent with previous studies. Several studies have found in the fields of nutrition, health, and body weight, women are often more conscientious than men.^(46,48)^ One study conducted on US students^(46)^ found that female participants had a significantly greater level of nutrition knowledge compared to male students.^(46)^ Von Bothmer & Fridlund (2005) reported female students maintained healthier eating habits and were more willing to change their dietary habits, compared to males who exhibited higher rates of overweight and obesity and showed a lower desire towards seeking nutrition advice and participating in health-enhancing activities.^(48)^ The current study identified that women were more likely to use nutrition labels, a finding confirmed by other studies.^(49,50)^ Therefore, to effectively improve nutrition knowledge and label use across the student population, nutrition promotion initiatives in Bangladeshi universities should adopt gender-sensitive strategies. These strategies should be designed to specifically engage male students, who demonstrated lower nutrition knowledge and label use, while also supporting and further enhancing the positive practices observed among female students.

Less than half of the students in our study reported high levels of dietary diversity, and students with satisfactory nutrition knowledge, and who frequently used nutrition labels, reported a more diverse diet than their counterparts. This aligns with previous research among school-age adolescents that found a positive association between adequate nutrition knowledge and diverse dietary habits.^(51)^ According to Omage & Omuemu (2018),^(52)^ individuals who possess greater knowledge of fundamental nutritional values across a variety of food groups tend to have more diversified dietary patterns.^(52)^ Furthermore, other studies have shown how regular nutrition label ‘users’ have a more diverse diet, display lower fat-related dietary behaviours, and higher fruit and vegetable consumption compared to those who rarely or never use nutrition labels.^(10)^ Simply, individuals who are more knowledgeable about nutrition may be making better food choices (i.e. avoiding unhealthy options) as a result of the nutritional information presented to them on products.^(44)^ Nutritional knowledge has the potential to influence eating habits and healthy lifestyles.^(53)^ However, it is crucial to understand that certain consumers may not have an interest in reading nutrition labels, regardless of their understanding of nutrition knowledge, and it must be noted that the present study did not explore this particular issue, and it is possible that consumer motivation plays an essential part in encouraging people to consider nutrition when making food choices (54). Collectively, these data support the notion that 1) irrespective of label use, nutrition knowledge is important in managing healthy dietary habits (10, 12, 55), and 2) nutrition awareness education that addresses and promotes the use of nutrition labels is a strategy that can influence dietary diversity, improve nutritional intake, and potentially contribute to a reduction in the prevalence of diet-related chronic diseases.

Among the surveyed students, the least consumed food group was milk and milk products, organ meat and vitamin A-rich fruits and vegetables. This may be explained by the majority of students in this study living in university halls and coming from middle-class families. The limited access to a variety of food options in university dining facilities may have led students to frequently opt for more affordable and readily available foods. Dissatisfaction with the limited variety and palatability of dining hall food has been reported by the Bangladeshi university students in previous studies.^(56,57)^ Additionally, these consumption patterns can be influenced by food taste, dietary preferences, cultural norms, and cost considerations.^(28)^ For instance, organ meat may be less favoured due to taste, while dairy and specific produce might be perceived as less affordable or less convenient. This interpretation is supported by the relatively higher consumption of other protein sources (like eggs and meat/fish/seafood) and other vegetable groups, indicating selective choice within available options. Therefore, while the overall dietary diversity remains a key indicator of nutritional adequacy, these specific gaps highlight a priority for nutrition promotion. Collectively, this study highlights the need for university dining facilities in Bangladesh to make a concerted effort to improve the availability of diverse and nutrient-rich food on campus, top optimally support the overall well-being and academic success of the students.

Globally, inadequate nutrition knowledge and low dietary diversity are serious matter in aspects of public health nutrition. Our findings revealed a large proportion of the university students had unsatisfactory nutrition knowledge and medium-to-low dietary diversity, indicating unique healthy diet barrier in the Bangladeshi context. There are several reasons to justify why nutrition knowledge and dietary diversity might be particularly low in this specific context. (i) Nutrition transformation exacerbates inadequate dietary diversity in Bangladesh by replacing the traditional balanced meals with processed, low-nutrient convenience foods. This transition results in a double burden – micronutrient deficiencies from insufficient diversity alongside increasing obesity risks from high-energy and processed modern meals. (ii) University students often depends on affordable but monotonous and repetitive diets (e.g. carbohydrate/rice-centric meals, limited fruits/vegetables) due to economic constraints and limited access to diverse foods in university campus cafeterias. (iii) Cultural preferences for energy-dense staples over micronutrient-rich meals continue, exacerbated by a dearth of nutrition education in school curriculum. (iv) Urban university surroundings may expose students to unhealthy dietary environments (e.g. street food, marketing of processed snacks), further reducing food diversity.

### Recommendations for policy and practice

This is one of the first studies to assess the prevalence and predictors of nutrition knowledge, nutrition label use, and dietary diversity among a large representative sample of public university students in Bangladesh. The findings can be used for developing and launching nutrition education programmes at the university level to increase nutrition knowledge and nutrition label use among students. The Government of Bangladesh, specifically through the Ministry of Education in formal collaboration with the Ministry of Health and Family Welfare, could adopt a policy to mandate the integration of a standardized nutrition education module into the core curriculum of all public universities. This policy would provide the necessary directive and resources to ensure systematic nationwide implementation. At the institutional level, public universities can translate these policy priorities into practical actions. These include 1) implementing structured nutrition education initiatives such as curriculum-linked modules, 2) orientation-based awareness sessions to junior or newly enrolled students, and/or 3) peer-led programmes to improve nutrition knowledge and encourage the use of nutrition labels.

The authors suggest the following three context-specific interventions (informed by existing literature) to promote nutrition knowledge and dietary diversity among Bangladeshi university students: (i) integrating practical nutrition education into existing university curricula or extracurricular programmes could address significant gaps, with content tailored to traditional food habits and fundamental of food and nutrition (e.g. affordable plant-based protein sources like lentils and chickpeas, or seasonal fruits/vegetables availability),^(58–60)^ (ii) social media campaigns to counter common misconceptions regarding food costs (e.g. ‘healthy eating is expensive’),^(61,62)^ and (iii) working with campus vendors to increase access to diverse meals, nutritious options including fortified snacks and vegetable-based meals.^(61,63–66)^ Additionally, integrating nutrition counselling services inside university health facilities could provide personalized dietary advice. Together, these multi-level initiatives address both individual knowledge gaps and systemic barriers, providing a culturally appropriate framework for improving student nutrition.

### Contribution to nutritional science

This study extends previous research by focusing on a young, educated demographic often believed to have better nutrition education and knowledge. Contrary to expectations, significant gaps were identified among Bangladeshi university students, suggesting that education alone may not overcome structural and cultural dietary constraints. This finding challenges a common assumption and underscores the need for targeted food and nutrition literacy initiatives regardless of educational attainment. Furthermore, our study provides key evidence by establishing that satisfactory nutrition knowledge and frequent nutrition label use are independently associated with higher dietary diversity. These initial associations offer an evidence-based model for designing effective, multi-component interventions to improve diet quality in similar settings. Adopting a holistic approach grounded in this evidence can generate actionable insights, facilitating the development of targeted educational strategies that enhance nutrition knowledge, promote informed food choices, and ultimately improve dietary diversity among young adults in Bangladesh.

### Limitations

This study was not without limitations. First, the study design was cross-sectional in nature, therefore we cannot make causal inferences from our data. Second, this study recruited participants from the common campus areas of the purposefully selected public universities across three divisions, may narrow the generalizability of the findings. While this recruitment approach ensured feasibility, generalizing these findings to all Bangladeshi university students, particularly those from private institutions, other areas, or different academic settings need careful consideration. Third, although study major was adjusted in the regression models, inherent differences in nutritional knowledge between health-related and non-health-related disciplines may affect the study’s generalizability. Fourth, although validated and standard approaches were used for assessing nutrition knowledge and dietary diversity, reliance on self-reported measures (including BMI, physical activity, nutrition label use, food consumption patterns, etc.) can be subject to recall and social desirability biases, which may affect the accuracy of our findings. In addition, using the single-item measure for nutrition label use was another limitation, as it may not capture the broad concept of nutrition labelling behaviours. Future research could incorporate validated multi-item scales or mixed-method approaches to enhance reliability.

## Conclusion

Most university students in this study reported unsatisfactory levels of nutrition knowledge, with female respondents reporting higher levels of nutrition knowledge and more frequent use of nutrition labels compared to males. Participants with satisfactory nutrition knowledge and frequent use of nutrition labels had a higher dietary diversity compared to their counterparts. These results emphasize the importance of implementing programmes that target young adults that specifically address nutrition knowledge and nutrition label education to allow individuals to make informed and healthy choices in relation to their dietary practices. Public health professionals, policymakers, and nutritionists should prioritize the development of strategies that can be accessible to all to improve the dietary patterns and the nutritional status of young Bangladeshi adults. Further research is warranted that longitudinally examines the relationship between nutrition knowledge and nutrition label use with dietary diversity among university students and young people in Bangladesh.
